# The Hospital

**Published:** 1917-11-10

**Authors:** 


THE
### INSTITUTIONAL WORKER
**Being a Special Supplement to "The Hospital"**

### OUR BUREAU OF INFORMATION.

### Rules for Correspondents.

1. Every letter must be accompanied by the coupon to be cut from the back cover (inside page) of The Hospital, current issue, and must contain the name and address of the correspondent with pseudonym for publication if desired. Replies by post cannot be given save under exceptional circumstances at the Editor's discretion.

2. Letters from Approved Homes in reply to special needs published in the Bureau should state terms and full particulars, and be sent prepaid under cover to the Editor of the Bureau with name written across coupon for identification.

3. Proprietors of Homes which have not yet been entered on the List of Approved Homes, but have spare accommodation likely to suit special needs, are invited to write for an application form for registration. The fee for registration, which includes two announcements of the Home in the Bureau and other privileges, is 10s.

4. All communications to be addressed to the Editor of The Hospital, 28 Southampton Street, Strand, London, W.C. 2, and marked "Bureau of Information."

### PATRIOTIC CHRISTMAS COOKERY.

### The Ministry of Food's Recipes.

At a demonstration of patriotic Christmas cookery which was held under the auspices of the Ministry of Food at Grosvenor House on November 2, some very useful and economical recipes were given. As Sir Arthur Yapp, the Director of Food Economy, pointed out, it is of the utmost importance that still stricter economy should be observed in the use of dried fruits, such as currants, sultanas, and raisins, for three reasons: firstly, they are in themselves valuable foodstuffs; secondly, they are useful for sweetening purposes, and will to a certain extent reduce the demand for sugar; and thirdly, owing mainly to tonnage difficulties, the available supplies of dried fruit this season will be restricted. At the same time, the Ministry feels that a little extra is required at Christmastime, and with this in view it has, with the help of Mons. Cedard, the King's chef, Mr. Herbert Senn, and others, formulated some excellent recipes for Christmas puddings and mincemeat. All the recipes are wholesome and satisfying, although simple and much less costly than those of pre-war Christmases. They ought, therefore, to be most welcome to institutional housekeepers, who are already wondering how they are to give their patients Christmas fare and at the same time practise economy. There are three recipes to choose from for Christmas puddings, varying in quality and cost.

*Recipe No. 1.*

This consists of 4 oz. of flour, 2 oz. of bread, 1 oz. chopped suet, 1 oz. currants, 2 oz. grated carrot, 2 oz. grated potato, ½ teaspoonful ground ginger, ½ teaspoonful of cinnamon, ½ teaspoonful carbonate of soda, ½ an egg, ½ gill milk, 1 oz. treacle. The cost of these ingredients works out at 7d., and the quantities are sufficient for a pudding for six persons.

Put all the dry ingredients into a mixing bowl. Mix the treacle or syrup with the milk, add the soda, and allow it to dissolve; then stir this into the dry ingredients (dried eggs are used). Mix all well together, and put into one or two greased pudding-basins. Cover with a cloth or greased paper, and boil or steam for fully three hours.

*Recipe No. 2.*

4 oz. flour, 2 oz. oatmeal, 2 oz. ground rice, 3 oz. fat, ½ teaspoonful salt, 1 dessertspoonful mixed spice, 2 oz. sultanas, 2 oz. mixed chopped peel, ½ lb. apples, 2 oz. grated carrot, 1 egg (dried), ½ gill milk, 2 oz. treacle, grated rind and juice of ½ lemon, ½ teaspoonful of baking-powder. The cost of these materials is 1s. 4½d., and the quantities are sufficient for six persons.

Put all the dry ingredients, previously prepared, into a large basin. Mix the baking-powder with the milk and syrup and egg; mix this into the dry ingredients and stir well. Put the mixture into greased basins and cover them with a cloth or greased paper. Boil for 2½ hours or steam for 3½ hours. Serve on a hot dish with sauce or custard.

*Recipe No. 3.*

4 oz. flour, 4 oz. soaked bread, 6 oz. chopped suet, ¼ teaspoonful salt, 1 dessertspoonful mixed spice, 4 oz. sultanas, 2 oz. mixed chopped peel, ½ lb. apples, 2 oz. grated carrot, 1 egg (dried), ½ gill milk, 2 oz. treacle, and the grated rind and juice of half a lemon. The cost of these ingredients is 1s. 8¾d., and the quantities are sufficient for six persons.

Weigh out and measure all the ingredients, prepare the dry materials and put them in a mixing bowl; stir all well together, then add the egg and milk. When thoroughly mixed, put the mixture into two well-greased pudding-basins, cover each with a cloth, and boil or steam for fully three hours.

It is suggested that the keeping quality of these puddings may not be so great as puddings of a richer kind, so they should therefore not be made too long before Christmas.

*Mincemeat.*

The mincemeat recipe is an excellent one, and works out at 2s. 3½d. for thirty-six mince-pies. The ingredients required are: 1¼ lb. apples, 6 oz. grated suet, ¼ lb. currants and raisins, ¼ lb. moist sugar or corn syrup, ¼ lb. figs, dates, or prunes (stoned), ¼ lb. candied peel, 1 oz. ground sugar, 1 oz. mixed spice, 1 lemon or orange, 1 gill of cider (optional). Peel and chop the apples, chop the dates, figs, or prunes, and candied peel. Clean the currants and raisins, mix all together.

*Short-crust Paste for Mince-pies.*

¼ lb. ordinary flour, 2 oz. maize flour, 2 oz. barley flour or cornflour, 4 oz. lard, dripping, or margarine, a pinch of salt, ½ teaspoonful bi-carbonate of soda, sufficient water to mix. The cost of the crust, sufficient for twelve pies, would be 6¾d.

Mix the flour, salt, and soda, and rub the fat into flour. Mix to a stiff paste with water and roll out.

### An Interesting Experiment.

What the Bedfordshire County Council describes as "an interesting experiment" has been started at Biggleswade Council Girls' School. Through the kindness and assistance of Dr. Welch, a practical lesson in washing and dressing a baby was given to the girls in the upper class by Nurse Stephens, the county health visitor of the district. The demonstration was followed by oral and written lessons on the subject as useful revision exercises. The Government Inspector has expressed his warmest admiration of the innovation, and if the sub-committee raises no objection, it is hoped to arrange for an extension of the work. The Chairman of the Notification of Births and Midwives Act Committee is of opinion that these demonstrations will have a beneficial influence on infant-welfare work, and is willing to allow occasional demonstrations to be given at suitable schools.

### Questions for November.

**1. The Equipment of an Out-Patient Department.**

State what you consider necessary for the general equipment of a new Out-Patient Department and Accident Room, and the procedure to be adopted for the provision of such requirements.

**2. How to Organise a Mothers' and Babies' Welcome.**

Outline a simple scheme which a midwife working independently and single-handed in a scattered country district could adopt to start a "Mothers and Babies' Welcome."

### RULES.

The following rules must be observed:—

1. Contributions must be written on one side of the paper. Brevity and terseness are desirable features. The MSS. must bear the name and address of the sender and be accompanied by coupon to be cut from the back cover (inside page) of the current issue of THE HOSPITAL. A pseudonym must be chosen if the name is not to be published.
2. Contributions must be addressed to the Editor of THE HOSPITAL, Institutional Worker Supplement, 28 & 29 Southampton Street, Strand, London, W.C. 2, should reach him before the end of the current month, and be marked in the left-hand corner "Facts and Figures."

**A minimum payment of Half-a-Guinea will be made for each published answer.**

### QUESTIONS INVITED.

IN connection with our Question Box, we offer every month five shillings each for the two best questions which are sent in for consideration. The questions may be on any institutional subject and concern any department, from the secretary's to the porter's, or the matron's to the domestic staff; the one essential is that they must be practical and deal with points that have a definite relation to hospital or institutional administration, upkeep, finance, or management. Points relating to artificers' work, housekeeping, the laundry, out-patients, and other practical matters will be welcome. It is hoped that thus, when difficult or doubtful points arise in the course of their work, institutional workers will be encouraged to put them in the form of a question and send them to the Editor, THE HOSPITAL Bureau of Information, 28 Southampton Street, Strand, W.C. 2, marked "Question Box."

The best questions will be published in due course and our readers will be given the opportunity of answering them. Thus all institutional workers may be able to co-operate with the view of helping the work and smoothing the difficulties of each other. In short, we want the Question Box of the *Institutional Worker* to be freely used by every worker habitually.

### ENQUIRIES AND ANSWERS.

### *THE SICK AND IN NEED.*

**Admission to Provincial Fever Hospitals.**

ADMISSION to a provincial fever hospital would be most readily obtained by communicating with the medical officer of health in your district. With regard to the cost of maintenance, the local sanitary authorities have the power to recover it from the patient, but usually (more especially if the patient is very poor) this power is not exercised, and the cost is thrown on to the rates. It is usually possible to make arrangements for special accommodation at the larger institutions.—H. W. G.

**Treatment of Tuberculosis.**

Walker Gate Fever Hospital, Newcastle-upon-Tyne, is one of the hospitals which have been approved by the Local Government Board under the Insurance Act of 1911 for the treatment of tuberculosis. We suggest that you write to the medical superintendent.—GORDON.

**Convalescent Home in Bournemouth.**

CHILDREN over twelve years of age are eligible for admission to the Herbert Convalescent Home, Westbourne, Bournemouth. A recommendation from a subscriber is necessary, otherwise a payment of 12s. 6d. is asked for. Application for admission should be made to the Secretary. If the girl is over fifteen she might possibly be admitted to the St. Joseph's Convalescent Home, Branksome Wood Road, Bournemouth. The terms of admission are by subscriber's letter or payment of 8s. per week. Washing is extra.—PETER.

**Convalescent Home for Mother and Infant.**

THE Convalescent Home for Women and Children, Limpsfield, Surrey, accepts mothers with their infants during the winter months on payment of 12s. 6d. per week. Yes, cases from London are received.—H. H.

### *EMPLOYMENT AND TRAINING.*

**District Nursing Society in South Australia.**

THERE is the District Trained Nursing Society of South Australia, 48 Old Exchange, Pirie Street, Adelaide. Certified nurses only are employed, and receive a salary of £100 to £110 per annum, with uniform. Write to the Lady Superintendent and Hon. Secretary.—FLORENCE.

**Training as District Nurse and Midwife.**

YOU no doubt refer to the Bedfordshire Rural Nursing Association. The Association was formed to provide nurses for the villages in Bedfordshire. There is a Central Fund in connection with the Association which gives help in training the nurses, and aid to poor districts when necessary. Candidates are required to serve for three years from completion of their training, and receive 13s. per week for the first year, 14s. the second, and 15s. for the third year, together with uniform and boots. Selected candidates are sent for nine months' midwifery, monthly, and district nursing to the Plaistow Maternity Home, Victoria Docks, E., and they become certified midwives. The Secretary's address is Lansdown House, Bedford.—ANXIOUS TO TRAIN.

### *MISCELLANEOUS.*

**X-Ray Tube Stand.**

AMONG the many pieces of scientific apparatus almost daily designed is an improved *x*-ray tube stand (the work of Messrs. Harry W. Cox and Co., Ltd., of Great Portland Street, London, W.), which has several excellent features meriting the attention of radiographers. The stand is equipped for both radiography and therapeutic work; it is perfectly stable and rigid. The tube box is efficiently protected, and provides a simple and reliable method for centring the anode; it has a stereoscopic movement both horizontally and vertically. The box has universal movement, the angle of which is capable of measurement. A unique feature is the mechanism for a stereoscopic anode tilt; this enables the radiographer to obtain a good stereoscopic picture when using a small extension tube. For treatment work metal compressors of various diameters are provided, and the pastille-holder is fixed at a distance from the anode which allows of a full dose being administered when the pastille reaches the half "B" tint. This is regarded by some radiologists as an advantage, because it is easier to judge the half tint than the full tint. Furthermore, the danger of an overdose is lessened, for the pastille changes colour very slowly after the full "B" tint is reached. The price of the stand is £35.—X-RAY.

**Handbook on Nursing in Common Ailments and Accidents.**

"THE ABC of Nursing in Accidents and Illnesses," by E. M. Clarke, may be of use to you. The price is 1s. net., and it is published by The Scientific Press, Ltd., 28 and 29 Southampton Street, Strand, W. C. 2.—A. B.

**Convalescent Homes' Association.**

THE address of the Convalescent Homes' Association is Denison House, Vauxhall Bridge Road, S.W. The objects of the Association are to co-ordinate the work of public convalescent homes receiving London convalescent patients and to obtain and disseminate information on the needs of London in respect of convalescent relief. Inquiries should be made of the Secretary. The inquiry fee is 1s. —SUPERINTENDENT.

